# Utilidade Diagnóstica da Duração da Onda P na Identificação de Sobrecarga Atrial Esquerda: Lições da Coorte Elsa-Brasil

**DOI:** 10.36660/abc.20250151

**Published:** 2025-11-26

**Authors:** Ana Cecília de Sena Oliveira, Vinicius Tostes Carvalho, Marcelo Martins Pinto-Filho, Luisa Caldeira Brant, Sandhi Maria Barreto, Murilo Foppa, Antonio Luiz Pinho Ribeiro

**Affiliations:** 1 Faculdade de Medicina Universidade Federal de Minas Gerais Belo Horizonte MG Brasil Faculdade de Medicina - Universidade Federal de Minas Gerais (UFMG), Belo Horizonte, MG – Brasil; 2 Universidade Federal do Rio Grande do Sul Porto Alegre RS Brasil Universidade Federal do Rio Grande do Sul (UFRS), Porto Alegre, RS – Brasil

**Keywords:** Eletrocardiografia, Cardiomiopatias, Átrios do Coração, Ecocardiografia

## Abstract

**Fundamento:**

A cardiomiopatia atrial abrange diversas anormalidades atriais, incluindo a sobrecarga atrial esquerda (SAE), que está associada a desfechos cardiovasculares e neurocognitivos adversos. A duração da onda P ≥ 120 ms no eletrocardiograma (ECG) de 12 derivações é uma ferramenta amplamente disponível e econômica, além de ser um dos principais critérios diagnósticos adotados. No entanto, seu desempenho diagnóstico em estudos de larga escala ainda é pouco explorado.

**Objetivos:**

Avaliar a correlação entre a duração da onda P e o volume do átrio esquerdo (VAE) medido pelo ecocardiograma transtorácico (ETT) e determinar a precisão diagnóstica da duração da onda P ≥120 ms para identificar a SAE.

**Métodos:**

Realizamos uma análise transversal dos dados basais dos participantes da coorte ELSA-Brasil em ritmo sinusal. A duração da onda P foi medida automaticamente por meio de ECG padrão de 12 derivações, com ≥120 ms indicando sobrecarga atrial. A SAE foi definida como VAE>34 mL/m^2^ por ETT. O teste de Spearman avaliou a correlação. Métricas de desempenho diagnóstico - sensibilidade (Sn), especificidade (Sp), valor preditivo positivo (VPP) e valor preditivo negativo (VPN), razão de verossimilhança positiva e negativa (RV+ e RV-) - foram calculadas. Além disso, foi construída uma Curva Característica de Operação do Receptor (ROC). A significância estatística foi estabelecida em valor de p<0,05 com intervalo de confiança de 95%.

**Resultados:**

O estudo incluiu 2.589 participantes, 419 (16,1%) com índice de VAE > 34 mL/m^2^. A duração da onda P correlacionou-se fracamente com o VAE (ρ de Spearman = 0,120, p < 0,001). As medidas de acurácia diagnóstica foram: AUC = 0,557 ± 0,032; Se = 0,296; Sp = 0,811; VPP = 0,232; VPN = 0,856; RV + 1,566; RV - 0,868.

**Conclusão:**

A duração da onda P demonstra valor diagnóstico limitado para identificar SAE, o que sugere que não é recomendado como critério diagnóstico isolado para essa condição.

## Graphical abstract

**Central Illustration:**
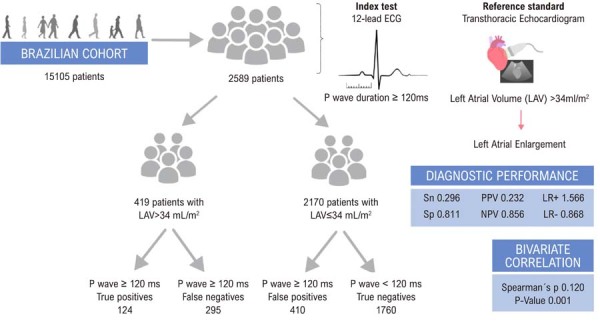
Diagnostic Utility of P-Wave Duration in Identifying Left Atrial Enlargement: Insights from the ELSA-Brasil Cohort

ECG: electrocardiogram; Sn: sensibility; Sp: specificity; PPV: positive predictive value. NPV: negative predictive value; LR+: positive likelihood ratio; LR-: negative likelihood ratio; LAV: Left Atrial Volume. Source: Illustration elaborated by the authors.

## Introdução

Cardiomiopatia atrial é qualquer alteração estrutural, arquitetônica, contrátil ou eletrofisiológica que afeta os átrios, com potencial para produzir manifestações clinicamente relevantes. Ela abrange diversas alterações, incluindo a sobrecarga atrial esquerda (SAE).^1,2^ A SAE, juntamente com a fibrose miocárdica, causa disfunção do átrio esquerdo (AE) e atraso na condução, resultando em remodelamento do AE, que é um substrato subjacente para desfechos cardiovasculares, como fibrilação atrial (FA) e acidente vascular cerebral (AVC).^3^ A presença de FA está associada a um aumento de 5 a 6 vezes no risco de AVC,^4^ enquanto AEs maiores aumentam o risco de AVC, mesmo em pacientes em ritmo sinusal.^5^

Portanto, a avaliação do tamanho e da anatomia do AE é fundamental para identificar remodelamento estrutural e caracterizar a miocardiopatia atrial. O método de avaliação preferencial para o tamanho do AE é a ecocardiografia,^3^ que pode não estar disponível, principalmente em locais com poucos recursos.

O eletrocardiograma (ECG) é uma ferramenta clínica de baixo custo e amplamente disponível usada para diagnosticar SAE analisando diversas anormalidades da onda P, uma vez que a SAE geralmente envolve o prolongamento do tempo de ativação atrial. Portanto, ela tende a causar ondas P de pico duplo ou entalhadas e aumentar a duração da onda P.^6^ No entanto, os critérios diagnósticos eletrocardiográficos para SAE têm desempenho limitado. A duração da onda P, em particular, demonstrou ter sensibilidade (Sn) notavelmente reduzida.^7-10^ De fato, um estudo sobre as faixas normais das medições de ECG em um grande conjunto de dados de pacientes de cuidados primários mostrou que o valor mediano da onda P é superior a 110 ms para homens com mais de 50 anos e mulheres acima de 60 anos, com uma proporção significativa de indivíduos com valores acima do limiar de 120 ms.^11^ No entanto, uma duração da onda P ≥ 120 ms na derivação D2 ainda é listada como um critério para SAE em diretrizes^12^ e livros didáticos.^13^

Apesar disso, estudos em humanos com grandes amostras que avaliem o desempenho diagnóstico da duração da onda P em SAE são escassos. Para preencher essa lacuna na literatura, analisamos dados de uma grande coorte brasileira, projetada para estudar os determinantes de doenças cardiovasculares em adultos, verificando a correlação entre a duração da onda P medida no ECG de 12 derivações e o volume do AE (VAE) no ecocardiograma transtorácico (ETT), bem como a precisão diagnóstica da duração da onda P superior a 120 ms para diagnosticar SAE (Ilustração Central).

## Métodos

Este estudo diagnóstico envolveu uma análise transversal de um subconjunto de participantes da coorte ELSA-Brasil. O ELSA-Brasil foi aprovado pelos Comitês de Ética em Pesquisa das instituições de ensino e pesquisa participantes (COEP - UFMG, número ETIC 186/06) e pela Comissão Nacional de Ética em Pesquisa (CONEP 976/20060) do Ministério da Saúde. Seguimos as diretrizes do *Standards for Reporting of Diagnostic Accuracy* (STARD).

### Pacientes

O ELSA-Brasil é um estudo de coorte multicêntrico que visa avaliar os determinantes de doenças cardiovasculares e diabetes em adultos brasileiros. A coorte original incluiu 15.105 servidores públicos de 5 universidades e um instituto de pesquisa, residentes em 6 cidades brasileiras (Belo Horizonte, Rio de Janeiro, São Paulo, Porto Alegre, Salvador e Vitória).^14^ A avaliação inicial foi realizada entre 2008 e 2010, durante a qual os participantes foram submetidos a entrevistas abrangentes e a exames clínicos e laboratoriais para coletar dados relativos a vários domínios fisiológicos, particularmente à saúde cardiovascular, bem como dados antropométricos e epidemiológicos. Todos os pacientes incluídos foram submetidos a um ECG de 12 derivações. Um ecocardiograma abrangente foi realizado em todos os participantes com mais de 60 anos no início do estudo, bem como em uma subamostra de 10% de pessoas selecionadas aleatoriamente entre 35 e 59 anos.

Para a análise atual, os participantes da avaliação basal (2008-2010) do estudo ELSA-Brasil foram considerados elegíveis se atendessem aos seguintes critérios de inclusão: (1) realização de ecocardiograma e ECG no momento da inclusão no estudo. Os critérios de exclusão foram os seguintes: (1) ausência de medida da onda P nos registros de ECG; (2) ausência de medida do VAE nos registros de ecocardiograma; (3) presença de fibrilação atrial e/ou flutter atrial; (4) frequência cardíaca abaixo de 50 bpm ou acima de 100 bpm; (5) presença de marcapasso ou válvula protética.

### Métodos de teste

O teste índice foi a duração da onda P medida automaticamente por meio de ECG padrão de 12 derivações obtido durante exames basais. Em nosso estudo, uma onda P igual ou maior que 120 ms foi considerada aumentada e diagnóstica de SAE.^1,6^ O ECG foi realizado de acordo com um protocolo previamente definido, utilizando um dispositivo digital (Atria 6100, Burdick, Cardiac Science Corporation, Bothell, WA, EUA), calibrado a 10 mm/mV e velocidade de 25 mm/segundo, com leitura automática da frequência cardíaca, duração, amplitude e eixos da onda P. As medições da onda P foram realizadas na derivação DII.^15,16^ Os ECGs registrados foram transmitidos para um banco de dados eletrônico do ECG Reading Center, criado para garantir a qualidade dos registros e sua codificação.^15^ Os ECGs foram submetidos à leitura posterior pelo programa de análise de ECG de Glasgow^17^ e à codificação pelo Código de Minnesota.^18-20^

O padrão de referência para aumento do AE foi um VAE superior a 34 mL/m^2^, avaliado por ecocardiografia transtorácica (ETT). O VAE foi calculado pela fórmula de Simpson e indexado pela Área de Superfície Corporal.^3,21^ A ecocardiografia transtorácica (Aplio XG; Toshiba Corporation, Tóquio, Japão) foi realizada por ecocardiografistas treinados. Imagens de cineloops e imagens estáticas de três ciclos cardíacos em ritmo cardíaco regular foram selecionadas seguindo um protocolo padrão baseado nas recomendações para o uso da ecocardiografia em pesquisas por ocasião do exame basal. Essas imagens foram então transmitidas e lidas em uma central de leitura, acompanhadas de um formulário detalhando a qualidade dos exames e descrições dos achados preliminares. Todos os estudos foram analisados e quantificados no centro de leitura ecocardiográfica do estudo, utilizando a mesma estação de trabalho (ComPACS 10.5; Medimatic SrL., Itália). Essa análise foi conduzida por três ecocardiografistas certificados que seguiram o protocolo de leitura estabelecido.^14,22^

### Análise estatística

Estatísticas descritivas foram utilizadas para resumir as características da população estudada, e uma análise estratificada por sexo foi realizada. As variáveis contínuas não apresentaram distribuição normal e foram expressas como medianas e intervalos interquartis (IIQ). Frequências e porcentagens relataram variáveis categóricas. A normalidade da distribuição foi avaliada pelo teste de Shapiro-Wilk.

A relação entre a duração da onda P e o VAE foi avaliada por meio de correlação bivariada. Considerando que ambas as variáveis eram contínuas e não seguiam uma distribuição normal, o teste não paramétrico de Spearman foi considerado apropriado. Um teste bicaudal foi realizado para avaliar a presença de correlações positivas e negativas. As comparações entre as medianas foram feitas com o teste U de Mann-Whitney. A significância estatística foi definida como um valor de p < 0,05 e um intervalo de confiança de 95%.

A acurácia diagnóstica foi avaliada utilizando uma tabela de contingência 2x2 para calcular Sn, especificidade (Sp), valor preditivo positivo (VPP), valor preditivo negativo (VPN), razão de verossimilhança positiva (RV+) e razão de verossimilhança negativa (RV-). Também construímos uma curva Característica de Operação do Receptor (ROC) para avaliar a área sob a curva (AUC). O VAE foi utilizado como padrão de referência (variável de estado). A duração da onda P medida no ECG serviu como variável de teste.

Todas as análises foram conduzidas usando o IBM SPSS Statistics, versão 21.

## Resultados

**Figura 1:**
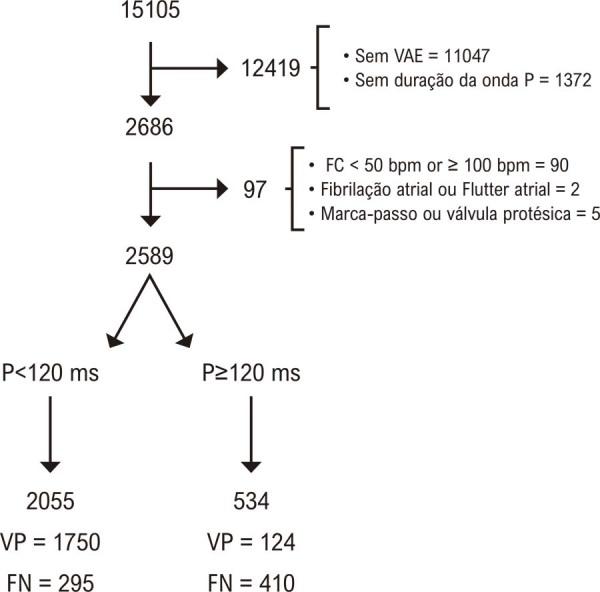
– Fluxograma ilustrando seleção de pacientes e taxas de falso positivo e falso negativo. FC: frequência cardíaca; VAE: volume do átrio esquerdo; VN: verdadeiro negativo; FN: falso negativo; VP: verdadeiro positivo; FP: falso positivo. Fonte: figura elaborada pelos autores.

O subconjunto original da Coorte ELSA-Brasil era composto por 15.105 indivíduos. Desses indivíduos, 12.419 foram excluídos por não atenderem aos critérios de inclusão, conforme ilustrado na Figura 1. A amostra final incluiu 2.589 pacientes.

As características demográficas, eletrocardiográficas e ecocardiográficas estão descritas na Tabela 1, bem como as taxas de fatores de risco cardiovascular: hipercolesterolemia, diabetes, infarto do miocárdio e acidente vascular cerebral. Dada a diferença significativa na duração mediana da onda P entre os sexos entre indivíduos sem doença cardiovascular observada na coorte ELSA-Brasil,^16^ considerou-se pertinente analisar a correlação entre a duração da onda P e o VAE por sexo. Nossa análise revelou que a duração mediana da onda P foi de 110 ms (IIQ, 18 ms) para mulheres e 114 ms (IIQ, 18 ms) para homens, com diferença estatisticamente significativa (p < 0,001).

**Tabela 1:** – Características demográficas, clínicas, ecocardiográficas e eletrocardiográficas dos pacientes

Característica	N = 2589
Sexo feminino, n (%)	1449 (55,9%)
Idade (anos), mediana (IIQ)	62 (10)
Área de superfície corporal (m^2^), mediana (IIQ)	1,76 (0,28)
Onda P (ms), mediana (IIQ)	112 (18)
Frequência cardíaca (bpm), mediana (IIQ)	65 (12)
Volume do AE (Simpson) (mL/m^2^), mediana (IIQ)	25,7 (9,7)
Infarto agudo do miocárdio, n (%)	100 (3,8)
Diabetes mellitus, n (%)	595 (22,9)
Hipercolesterolemia, n (%)	1179 (45,5)
Acidente vascular cerebral, n (%)	53 (2)

IIQ: intervalo interquartil; AE: átrio esquerdo. Fonte: Tabela elaborada pelos autores.

**Figura 2:**
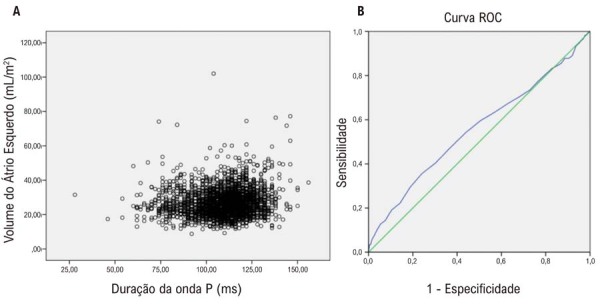
– A) Gráfico de dispersão (volume do átrio esquerdo X duração da onda P); B) Curva ROC: Curva Característica de Operação do Receptor. Fonte: figura elaborada pelos autores.

A duração da onda P correlacionou-se mal com o VAE no teste de correlação de Spearman. A correlação (ρ) entre a duração da onda P e o VAE foi de 0,120 (p < 0,001), e a área sob a curva ROC foi 0,557 ± 0,032 (Figura 2).

A tabela de contingência é apresentada na Tabela 2. Em nossa amostra, onde 419 (16,1%) apresentaram SAE, a duração da onda P ≥ 120 ms identificou corretamente 124 (23,2%) casos de SAE (Vol AE >34 mL/m^2^) e classificou incorretamente 410 casos (76,7%) como positivos. O mesmo limite para a duração da onda P foi observado em 1.760 casos verdadeiros negativos e 295 falsos negativos.

**Tabela 2:** – Tabulação cruzada da duração da onda P por medidas de volume do átrio esquerdo

	VAE > 34 ml/m^2^	VAE ≤ 34 ml/m^2^	
p≥120 ms	124 (29,6%)	410 (19%)	534
p<120 ms	295 (70,4%)	1760 (81,1%)	2055
Total	419	2170	2589

VAE: volume do átrio esquerdo. Fonte: Tabela elaborada pelos autores.

A Tabela 3 apresenta valores de desempenho diagnóstico e seus intervalos de confiança de 95%, mostrando o baixo desempenho da duração da onda P em discriminar o aumento do VAE. Quando estratificado por sexo, o desempenho diagnóstico permaneceu praticamente inalterado, com uma ligeira melhora na Sn observada entre os homens (0,366; IC de 95%, 0,300–0,437). Valores de corte alternativos de duração da onda P além de 120 ms foram avaliados para aumentar a Sp. No entanto, o aumento do limiar resultou em reduções substanciais na Sn (onda P ≥ 130 ms: Sn = 0,117, Sp = 0,952; onda P ≥ 140 ms: Se = 0,0285, Sp = 0,960). Por outro lado, as tentativas de melhorar a Sn levaram a perdas acentuadas de Sp (onda P ≥ 110 ms: Sn = 0,616, Sp = 0,461; onda P ≥ 105 ms: Sn = 0,705, Sp = 0,319).

**Tabela 3:** – Desempenho diagnóstico da duração da onda P >120 ms para o diagnóstico de dilatação atrial esquerda

Prevalência de SAE, n (%)	Sn (IC 95%)	Sp (IC 95%)	VPP (IC 95%)	VPN (IC 95%)	RV+ (IC 95%)	RV- (IC 95%)
419 (16,1%)	0,296 (0,254-0,341)	0,811 (0,794 - 0,827)	0,232 (0,198 - 0,270)	0,856 (0,841 - 0,871)	1.566 (1,319 -1,859)	0,868 (0,813 - 0,927)

SAE: sobrecarga atrial esquerda; Sn: sensibilidade; Sp: especificidade; VPP: valor preditivo positivo; VPN: valor preditivo negativo; RV+: razão de verossimilhança positiva; RV-: razão de verossimilhança negativa; IC: intervalo de confiança. Fonte: Tabela elaborada pelos autores.

## Discussão

Neste estudo transversal, demonstramos que a duração isolada da onda P apresenta fraca associação com o VAE e que uma duração da onda P ≥ 120 ms apresenta acurácia diagnóstica limitada para o reconhecimento de SAE, definida por VAE > 34 mL/m^2^. Apesar de sua Sp de moderada a alta, encontramos valores de Sn muito baixos, levando a taxas elevadas de falsos negativos. Nossos resultados também mostraram um VPP significativamente baixo, resultando em uma taxa elevada de testes falso-positivos: nesta amostra, 76% dos indivíduos com duração aumentada da onda P apresentavam VAE normal (Figura Central).

Nossos achados são consistentes com as evidências atualmente disponíveis, mostrando um desempenho diagnóstico limitado da duração da onda P para diagnosticar SAE, com valores de Sn moderados a baixos e valores de Sp moderados a altos. Estudos com delineamento semelhante constataram que os valores de Sn e Sp variaram, respectivamente, de 8% a 69% e de 49% a 90%.^7-10^ Até onde sabemos, nosso estudo incluiu o maior tamanho amostral de uma população miscigenada, visto que estudos anteriores relataram tamanhos amostrais variando de 71 a 551 participantes. Nossos resultados também corroboram a evidência de uma correlação inconsistente entre o ECG e os padrões de imagem de SAE, o que levou à introdução da terminologia de cardiomiopatia atrial.^1,23^

O aumento da taxa de testes falso-positivos observados ao considerar a duração da onda P ≥ 120 ms para o reconhecimento de SAE pode ser explicado por várias razões. Primeiro, ondas P maiores que 120 ms podem ser encontradas em indivíduos saudáveis. Um estudo de achados eletrocardiográficos na coorte ELSA-Brasil descobriu que as ondas P podem durar até 134 ms em homens e 130 ms em mulheres sem evidência de doença cardíaca clinicamente aparente.^16^ Além disso, durações prolongadas da onda P podem ser explicadas por variações fisiológicas e também ser induzidas por medicamentos. Durações prolongadas da onda P podem ser encontradas em atletas^24^ e indivíduos com maior índice de massa corporal.^25^ Além disso, alterações no sistema nervoso autônomo, como o bloqueio beta-adrenérgico, podem aumentar a duração da onda P.^16,26^ Finalmente, a duração da onda P pode ser aumentada por muitas condições patológicas além da SAE. Diabetes e neuropatia autonômica diabética também demonstraram estar associadas a durações mais longas da onda P.^27,28^ Tanto no bloqueio interatrial (BIA) parcial quanto no avançado, um atraso na condução através do feixe de Bachmann é uma causa bem conhecida da duração anormal da onda P. No entanto, no BIA avançado, podem ser encontradas alterações na morfologia da onda P, além do aumento da duração, como ondas P bifásicas (±) nas derivações II, III ou aVF. Negatividade final também pode ser observada na aVF, refletindo ativação retrógrada do AE.^1^

Considerando as evidências disponíveis, a duração da onda P não deve ser usada exclusivamente como critério diagnóstico de SAE. Além das elevadas taxas de falso-negativos, também produz inúmeras causas de resultados falso-positivos. Nesse cenário, o uso de uma onda P aumentada como critério diagnóstico suficiente para SAE pode levar ao sobrediagnóstico e a encaminhamentos inadequados para exames especializados, como o ecocardiograma.

Particularmente em cenários onde exames especializados e encaminhamentos para cuidados secundários são limitados, os encaminhamentos de pacientes sem anormalidades clínicas podem contribuir para longos tempos de espera e atrasos no diagnóstico.^29,30^ Nesses cenários, a implementação de novas ferramentas de triagem pode abrir caminho para a otimização dos encaminhamentos e a melhoria dos resultados em saúde. A importância do desenvolvimento dessas novas ferramentas de triagem também é enfatizada pela baixa Sn e pelos baixos valores de VPN desse parâmetro para o diagnóstico de SAE, visto que sua utilidade também é limitada na identificação de casos positivos.

A combinação de telecardiologia e ecocardiografia de triagem na atenção primária, por exemplo, parece ser uma estratégia promissora para estratificação de risco e predição de doenças cardíacas.^30^ Além disso, o uso de abordagens baseadas em inteligência artificial para detectar e prever doenças cardíacas com traçados de ECG é encorajador. Um modelo de rede neural convolucional apresentou excelente desempenho na detecção de doenças cardíacas estruturais em um estudo multinacional com pacientes únicos nos EUA e em uma coorte brasileira.^31^ Além disso, modelos baseados em aprendizado de máquina parecem ser um caminho promissor na compreensão dos parâmetros da onda P e da correlação entre padrões de imagem e eletrocardiográficos, bem como ferramentas valiosas no diagnóstico de SAE.^32,33^

Apesar de sua utilidade limitada para o diagnóstico de SAE, a duração da onda P, assim como outros parâmetros da onda P, está relacionada a desfechos de saúde mais precários, uma vez que pode traduzir patologia atrial além do aumento da câmara.^1^ Um estudo populacional que incluiu 8.561 indivíduos nos Estados Unidos descobriu que o aumento da duração da onda P se correlacionou com desfechos de mortalidade por todas as causas.^34^ Além disso, um estudo japonês com 810 indivíduos com um ou mais fatores de risco cardiovascular também descobriu que uma onda P prolongada (≥ 140 ms) estava independentemente associada a eventos cardíacos, independentemente de SAE.^35^ Uma duração prolongada da onda P também foi associada a risco elevado de FA,^36^ bem como a um risco aumentado de FA recorrente após ablação por cateter.^2^ No entanto, a utilidade da duração da onda P como um preditor de risco ainda é incerta e não pode ser entendida como um substituto para cardiomiopatia atrial, nem aplicada como uma ferramenta para influenciar decisões clínicas.^1^

Este estudo tem várias limitações. Primeiro, como uma análise secundária, não foi possível obter dados sobre parâmetros adicionais da onda P, como a força terminal da onda P em V1 ou a morfologia da onda P de pico duplo, impedindo uma avaliação de sua precisão diagnóstica combinada. Portanto, a análise combinada de outros parâmetros da onda P pode fornecer valores de Sn mais altos, que não foram avaliados neste estudo. Segundo, apenas 17% da coorte foi submetida a avaliação ecocardiográfica, potencialmente limitando a generalização de nossos achados para toda a coorte. Terceiro, nossa amostra incluiu indivíduos com idade entre 35 e 74 anos (70% deles com mais de 50 anos), o que limita a aplicabilidade de nossos achados a essa faixa etária específica e pode impedir sua generalização para populações mais jovens ou mais velhas. Finalmente, questões técnicas podem desempenhar um papel no aumento dos valores da onda P, uma vez que as medições digitais e da onda P podem variar entre métodos manuais e automatizados e estão sujeitas a erros.^1,37^

## Conclusão

A medição isolada da duração da onda P tem valor diagnóstico limitado para SAE, devido à sua baixa Sn e VPP, combinados com altas taxas de resultados falso-negativos. Consequentemente, não deve ser empregada como critério diagnóstico independente para SAE. No entanto, seu baixo desempenho diagnóstico para SAE, duração da onda P e outros parâmetros da onda P está cada vez mais associado a desfechos cardiovasculares e neurocognitivos adversos, devido à sua correlação com a miocardiopatia atrial. A capacidade preditiva dos parâmetros da onda P permanece uma área de pesquisa pouco explorada, com potencial promissor para o avanço da prática clínica.
