# Supplementary material for: Utilidade Diagnóstica da Duração da Onda P na Identificação de Sobrecarga Atrial Esquerda: Lições da Coorte Elsa-Brasil
Source: Arq Bras Cardiol. 2025 Nov 26;122(10):e20250151. [Article in Portuguese] doi: 10.36660/abc.20250151 (PMC12677763; doi:10.36660/abc.20250151)
Supplement: Translation

# Translation: Diagnostic Utility of P-Wave Duration in Identifying Left Atrial Enlargement: Insights from the ELSA-Brasil Cohort

**DOI:** 10.36660/abc.20250151i

**Authors:** Ana Cecília de Sena Oliveira, Vinicius Tostes Carvalho, Marcelo Martins Pinto-Filho, Luisa Caldeira Brant, Sandhi Maria Barreto, Murilo Foppa, Antonio Luiz Pinho Ribeiro

**Affiliations:** 1 Brazil Faculdade de Medicina - Universidade Federal de Minas Gerais (UFMG), Belo Horizonte, MG – Brazil; 2 Brazil Universidade Federal do Rio Grande do Sul (UFRS), Porto Alegre, RS – Brazil

**Keywords:** Electrocardiography, Cardiomyopathies, Heart Atria, Echocardiography

## Abstract

**Background:**

Atrial cardiomyopathy encompasses various atrial abnormalities, including left atrial enlargement (LAE), which is associated with adverse cardiovascular and neurocognitive outcomes. P-wave duration ≥120 ms on 12-lead electrocardiogram (ECG) is a widely available, cost-effective tool and one of the main diagnostic criteria adopted. However, its diagnostic performance in large-scale studies remains underexplored.

**Objectives:**

To evaluate the correlation between P-wave duration and left atrial volume (LAV) measured by transthoracic echocardiogram (TTE) and determine the diagnostic accuracy of P-wave duration ≥120 ms for identifying LAE.

**Methods:**

We conducted a cross-sectional analysis of baseline data from the ELSA-Brasil cohort participants in sinus rhythm. P-wave duration was automatically measured via standard 12-lead ECG, with ≥120 ms indicating enlargement. LAE was defined as LAV>34 mL/m^2^ by TTE. Spearman’s rank test assessed correlation. Diagnostic performance metrics - sensitivity (Sn), specificity (Sp), positive predictive value (PPV), and negative predictive value (NPV), positive and negative likelihood ratio (LR+ and LR-) - were calculated. Also, a Receiver Operating Characteristic (ROC) was constructed. Statistical significance was set at p-value<0.05 with 95% confidence interval.

**Results:**

The study included 2589 participants, 419 (16,1%) with LAV index>34 mL/m^2^. P-wave duration correlated weakly with LAV (Spearman’s ρ = 0.120, p < 0.001). Diagnostic accuracy measurements were AUC = 0.557 ± 0.032; Sn = 0.296; Sp = 0.811; PPV = 0.232; NPV = 0.856; LR+ 1.566; LR- 0.868.

**Conclusion:**

P-wave duration demonstrates limited diagnostic value for identifying LAE, suggesting against its recommendation as a standalone diagnostic criterion for this condition.

## Introduction

Atrial cardiomyopathy is any structural, architectural, contractile, or electrophysiological change affecting the atria with the potential to produce clinically relevant manifestations. It encompasses several alterations, including left atrial enlargement (LAE).^1,2^ LAE, along with myocardial fibrosis, causes LA dysfunction and conduction delay, resulting in LA remodeling, which is an underlying substrate for cardiovascular outcomes such as atrial fibrillation (AF) and stroke.^3^ The presence of AF is associated with a 5-6-fold increase in stroke risk,^4^ while larger LA sizes increase stroke risk, even in patients in sinus rhythm.^5^

Therefore, LA size and anatomy assessment are paramount to identifying structural remodeling and characterizing atrial cardiomyopathy. The preferred assessment method for LA size is echocardiography,^3^ which may be unavailable, particularly in low-resource settings.

The electrocardiogram (ECG) is a low-cost and widely available clinical tool used to diagnose LAE by analyzing several P-wave abnormalities since LAE usually involves prolonging atrial activation time. Therefore, it tends to cause double-peaked or notched P waves and increase P-wave duration.^6^ However, electrocardiographic diagnostic criteria for LAE have limited performance. P-wave duration, in particular, has been shown to have remarkably decreased sensitivity (Sn).^7-10^ Indeed, a study on normal ranges of the ECG measurements in a large dataset of primary care patients showed that the P wave median value is over 110 ms for men over 50 years old and women above 60 years old, with a significant proportion of subjects with values above the threshold of 120 ms.^11^ However, a P wave duration ≥ 120 ms in D2 lead is still listed as a criterion for LAE in guidelines^12^ and textbooks.^13^

Despite that, studies in humans with large sample sizes that appraise the P-wave duration diagnostic performance of LAE are scarce. To fill this gap in the literature, we analyzed data from a large Brazilian cohort designed to study the determinants of cardiovascular diseases in adults verifying the correlation between P-wave duration as measured in 12-lead ECG and LAV in the transthoracic echocardiogram (TTE), as well as the P-wave duration greater than 120 ms diagnostic accuracy to diagnose LAE (Central Illustration).

## Methods

This diagnostic study involved a cross-sectional analysis of a subset of participants from the ELSA-Brasil cohort. ELSA-Brasil was approved by the Research Ethics Committees of participating education and research institutions (COEP - UFMG, number ETIC 186/06) and by the National Research Ethics Committee (CONEP 976/20060) of the Ministry of Health. We followed the Standards for Reporting of Diagnostic Accuracy (STARD) reporting guidelines.

### Patients

ELSA-Brasil is a multicenter cohort study that aims to evaluate the determinants of cardiovascular disease and diabetes in Brazilian adults. The original cohort included 15,105 civil servants from 5 universities and one research institute, living in 6 Brazilian cities (Belo Horizonte, Rio de Janeiro, São Paulo, Porto Alegre, Salvador, and Vitória).^14^ Baseline assessment was conducted from 2008 to 2010, during which participants underwent comprehensive interviews and clinical and laboratory tests to collect data concerning several physiological domains, particularly cardiovascular health, as well as anthropometric and epidemiological data. All patients enrolled underwent a 12-lead electrocardiogram. A comprehensive TTE was done on all participants over the age of 60 at the start of the study, as well as on a randomly chosen 10% subsample of people aged 35 to 59.

For the current analysis, participants of the baseline assessment (2008-2010) of the ELSA-Brasil study were considered eligible if they met the following inclusion criteria: (1) completion of both an TTE and an electrocardiogram at the time of study inclusion. Exclusion criteria were as follows: (1) absence of P-wave measurement in ECG records; (2) absence of left atrial volume (LAV) measurement in TTE records; (3) presence of AF and/or atrial flutter; (4) heart rate below 50 bpm or over 100 bpm; (5) presence of a pacemaker or prosthetic valve.

### Test methods

The index test was the P-wave duration automatically measured via standard 12-lead electrocardiography (ECG) obtained during baseline examinations. In our study, a P-wave equal to or greater than 120 ms was considered enlarged and diagnostic of LAE.^1,6^ ECG was performed according to a previously defined protocol, using a digital device (Atria 6100, Burdick, Cardiac Science Corporation, Bothell, WA, USA), calibrated at 10 mm/mV and speed of 25 mm/second, with automatic reading of the heart rate, duration, amplitude, and axes of the P-wave. P wave measurements were performed in DII lead.^15,16^ Recorded ECGs were transmitted to an electronic database of the ECG Reading Center, which was created to secure the quality of records and their coding.^15^ ECGs underwent posterior reading by the Glasgow ECG analysis program^17^ and codification by the Minnesota Code.^18-20^

The reference standard for LAE was a LAV greater than 34 mL/m^2^, assessed by transthoracic echocardiography (TTE). LAV was calculated using Simpson’s formula and indexed to Body Surface Area.^3,21^ Transthoracic echocardiography (Aplio XG; Toshiba Corporation, Tokyo, Japan) was performed by trained echocardiographers. Cine loops and static images of 3 cardiac cycles under regular cardiac rhythm were selected following a standard protocol based on recommendations for using echocardiography in research on the occasion of baseline examination. These images were then transmitted to and read at a central reading center, accompanied by a form detailing the quality of the exams and descriptions of preliminary findings. All studies were analyzed and quantified at the study’s echocardiographic reading center using the same workstation (ComPACS 10.5; Medimatic SrL., Italy). This analysis was conducted by three certified echocardiographers who followed the established reading protocol.^14,22^

### Statistical analysis

Descriptive statistics were used to summarize the characteristics of the study population, and a sex-stratified analysis was performed. Continuous variables did not present a normal distribution and were summarized as medians and interquartile ranges (IQR). Frequencies and percentages reported categorical variables. The normality of the distribution was assessed using the Shapiro-Wilk test.

The relationship between P-wave length and LAV was assessed with a bivariate correlation. Given that both variables were continuous and did not follow a normal distribution, Spearman’s non-parametric test was deemed appropriate. A two-tailed test was performed to evaluate the presence of both positive and negative correlations. Comparisons between medians were made with Mann-Whitney U test. Statistical significance was defined as a p-value<0.05 and a 95% confidence interval.

Diagnostic accuracy was assessed using a 2x2 contingency table to calculate sensitivity (Sn), specificity (Sp), positive predictive value (PPV), negative predictive value (NPV), positive likelihood ratio (LR+), and negative likelihood ratio (LR-). We have also constructed a Receiver Operating Characteristic (ROC) curve to evaluate the area under the curve (AUC). LAV was used as the reference standard (state variable). P-wave duration measured at the ECG served as the test variable.

All analyses were conducted using IBM SPSS Statistics, version 21.

## Results

The original subset of the ELSA-Brasil Cohort comprised 15105 individuals. Of these individuals, 12419 were excluded because they did not meet the inclusion criteria, as illustrated in Figure 1. The final sample included 2589 patients.

**Figure 1:** – Flowchart illustrating patient selection, false positive and false negative rates. HR: heart rate; LAV: left atrial volume; TN: true negative; FN: false negative; TP: true positive; FP: false positive. Source: figure elaborated by the authors.

Demographic, ECG, and echocardiographic characteristics are described in Table 1, as well as rates of cardiovascular risk factors: hypercholesterolemia, diabetes, myocardial infarction, and cerebral vascular accident**.** Given the significant difference in the median P-wave duration between sexes among individuals without cardiovascular disease observed in the ELSA-Brasil cohort,^16^ it was considered pertinent to analyze the correlation between P-wave duration and LAV by sex. Our analysis revealed that the median P-wave duration was 110 ms (IQR, 18 ms) for females and 114 ms (IQR, 18 ms) for males, with a statistically significant difference (p<0.001).

**Table 1:** – Demographic, clinical, echocardiographic, and electrocardiographic characteristics of the patients

Characteristic	N = 2589
Female, n (%)	1449 (55.9%)
Age (years), median (IQR)	62 (10)
Body Surface Area (m^2^), median (IQR)	1,76 (0.28)
P wave (ms), median (IQR)	112 (18)
Heart Rate (bpm), median (IQR)	65 (12)
LAV (Simpson) (mL/m^2^), median (IQR)	25,7 (9.7)
Acute myocardial infarction, n (%)	100 (3.8)
Diabetes mellitus, n (%)	595 (22.9)
Hypercholesterolemia, n (%)	1179 (45.5)
Cerebral vascular accident, n (%)	53 (2)

IQR: interquartile range; LAV: left atrial volume. Source: Table elaborated by the authors.

P-wave duration correlated poorly with LAV in Spearman’s rank correlation test. The correlation (ρ) between P-wave duration and LAV was 0.120 (p < 0.001), and the area under the ROC curve was 0.557 ± 0.032 (Figure 2).

**Figure 2:** – A) Dispersion graph (Left Atrial Volume X P-wave duration); B) ROC curve: Receiver Operating Characteristic Curve. Source: figure elaborated by the authors.

The contingency table is provided in Table 2. In our sample, where 419 (16,1%) had LAE, P-wave duration ≥120 ms correctly identified 124 (23,2%) cases of LAE (LAV >34 mL/m^2^) and incorrectly classified 410 cases (76,7%) as positives. The same threshold for P-wave duration was observed in 1760 true negative and 295 false negative cases.

**Table 2:** – Cross-tabulation of P wave duration by Left Atrial Volume measurements

	LAV > 34 ml/m^2^	LAV ≤ 34 ml/m^2^	
p≥120 ms	124 (29.6%)	410 (19%)	534
p<120 ms	295 (70.4%)	1760 (81.1%)	2055
Total	419	2170	2589

LAV: left atrial volume. Source: Table elaborated by the authors.

Table 3 displays diagnostic performance values and their 95% confidence intervals, showing the poor performance of the P wave length in discriminating the increase in LAV. When stratified by sex, the diagnostic performance remained largely unchanged, with a slight improvement in Sn observed among males (0.366; 95% CI, 0.300–0.437). Alternative P-wave duration cut-off values beyond 120 ms were evaluated to enhance Sp. However, increasing the threshold resulted in substantial Sn reductions (P-wave ≥130 ms: Sn = 0.117, Sp = 0.952; P-wave ≥140 ms: Sn = 0.0285, Sp = 0.960). Conversely, attempts to improve Sn led to marked Sp losses (P-wave ≥110 ms: Se = 0.616, Sp = 0.461; P-wave ≥105 ms: Sn = 0.705, Sp = 0.319).

**Table 3:** – Diagnostic performance of P-wave duration >120 ms for the diagnosis of Left Atrial Enlargement

Prevalence of LAE, n (%)	Sn (95% CI)	Sp (95% CI)	PPV (95% CI)	NPV (95%CI)	LR+ (95%CI)	LR- (95%CI)
419 (16.1%)	0.296 (0.254-0.341)	0.811 (0.794 - 0.827)	0.232 (0.198 - 0.270)	0.856 (0.841 - 0.871)	1.566 (1.319 -1.859)	0.868 (0.813 - 0.927)

LAE: left atrial enlargement; Sn: sensibility; Sp: specificity; PPV: positive predictive value; NPV: negative predictive value; LAE: left atrial enlargement; LR+: positive likelihood ratio; LR-: negative likelihood ratio; CI: confidence interval. Source: Table elaborated by the authors.

## Discussion

In this cross-sectional study, we showed that isolated P-wave duration has a weak association with LAV and that a P-wave duration ≥120 ms has limited diagnostic accuracy for recognizing LAE defined by LAV >34 mL/m^2^. Despite its moderate to high Sp, we have found very low Sn values, leading to increased rates of false negatives. Our results also showed a significantly low PPV, yielding an elevated rate of false positive tests: in this sample, 76% of individuals with an increased P-wave duration had normal LAV (Central Illustration).

Our findings are consistent with the currently available evidence, showing a limited diagnostic performance of P-wave duration to diagnose LAE, with moderate to low Sn values and moderate to high Sp values. Studies with a similar design found that Sn and Sp values ranged, respectively, from 8% to 69% and 49% to 90%.^7-10^ To the best of our knowledge, our study included the largest sample size of a racially mixed population, as previous studies reported sample sizes varied from 71 to 551 participants. Our results also support the evidence of an inconsistent correlation between ECG and imaging patterns of LAE, which has led to the introduction of the atrial cardiomyopathy terminology.^1,23^

The increased rate of false positive tests observed when considering the P-wave duration ≥120 ms for recognizing LAE can be explained by several reasons. First, P-waves greater than 120 ms may be found in healthy individuals. A study of electrocardiographic findings in the ELSA-Brasil cohort found that P-waves could last up to 134 ms in males and 130 ms in females without evidence of clinically apparent heart disease.^16^ Additionally, prolonged P-wave durations can be explained by physiological variations and also be drug-induced. Prolonged P-wave durations can be found in athletes^24^ and individuals with greater body mass index.^25^ Also, alterations in the autonomic nervous system, such as beta-adrenergic blockade, can increase P-wave duration.^16,26^ Finally, P wave duration can be increased by many pathologic conditions other than LAE. Diabetes and diabetic autonomic neuropathy have also been shown to be associated with longer P-wave durations.^27,28^ In both partial and advanced interatrial block (IAB), a conduction delay through the Bachmann’s bundle is a well-known cause of abnormal duration of the P-wave. However, in advanced IAB, alterations in P-wave morphology other than increased duration can be found, such as biphasic (±) P-waves in leads II, III, or aVF. Final negativity can also be seen in aVF, reflecting retrograde activation of the LA.^1^

Given the available evidence, P-wave duration should not be used solely as a diagnostic criterion of LAE. In addition to its elevated false-negative rates, it also yields numerous causes of false-positive results. In this scenario, the use of an increased P-wave as a sufficient diagnostic criterion of LAE may lead to overdiagnosis and inappropriate referrals for specialized tests, such as a TTE.

Particularly in settings where specialized tests and secondary care referrals are limited, referrals of patients without clinical abnormalities may contribute to long waiting times and diagnostic delays.^29,30^ In these scenarios, implementing new screening tools may pave the way to optimizing referrals and improving health outcomes. The importance of the development of these new screening tools is also emphasized by the low Sn and low NPV values of this parameter to the diagnosis of LAE since its utility is also limited in identifying positive cases.

The combination of telecardiology and screening echocardiography in primary care, for instance, seems to be a promising strategy for risk stratification and prediction of heart disease.^30^ Also, the use of artificial intelligence based approaches to detect and predict heart disease with ECG tracings is encouraging. A convolutional neural network model had excellent performance in detecting structural heart disease in a multinational study with unique patients in the US and a Brazilian cohort.^31^ Additionally, machine-learning-based models seem to be a promising avenue in the understanding of P-wave parameters and the correlation between imaging and electrocardiographic patterns, as well as valuable tools in the diagnosis of LAE.^32,33^

Despite its limited utility for LAE diagnosis, P-wave duration, like other P-wave parameters, is related to poorer health outcomes since it may translate atrial pathology beyond chamber enlargement.^1^ A population-based study that included 8561 subjects in the United States found that increased P-wave duration correlated with all-cause mortality outcomes.^34^ Furthermore, a Japanese study with 810 subjects with one or more cardiovascular risk factors also found that a prolonged P-wave (≥ 140 ms) was independently associated with cardiac events, independently of LAE.^35^ A prolonged P-wave duration was also associated with elevated AF risk,^36^ as well as an increased risk of recurrent AF after catheter ablation.^2^ However, the usefulness of P-wave duration as a risk predictor is still uncertain and cannot be understood as a surrogate for atrial cardiomyopathy, nor applied as a tool to influence clinical decisions.^1^

This study has several limitations. First, as a secondary analysis, it was not possible to obtain data on additional P-wave parameters, such as P-wave terminal force in V1 or double-peaked P-wave morphology, precluding an evaluation of their combined diagnostic accuracy. Therefore, the combined analysis of other P-wave parameters may provide higher Sn values, which were not evaluated in this study. Second, only 17% of the cohort underwent echocardiographic assessment, potentially limiting the generalizability of our findings to the whole cohort. Third, our sample included individuals aged between 35 and 74 years old (70% of them aged over 50), which limits the applicability of our findings to this specific age range and may preclude their generalizability to younger or older populations. Finally, technical issues may play a role in increased P-wave values, since digital and P-wave measurements may vary between manual and automated methods and are subject to error.^1,37^

## Conclusion

Isolated P-wave duration measurement has limited diagnostic value for LAE, due to its low Sn and PPV, combined with high rates of false negative results. Consequently, it should not be employed as a standalone diagnostic criterion for LAE. Nevertheless, its low diagnostic performance for LAE, P-wave duration, and other P-wave parameters is increasingly associated with adverse cardiovascular and neurocognitive outcomes, due to its correlation with atrial cardiomyopathy. The predictive capacity of P-wave parameters remains an underexplored area of research with promising potential for advancing clinical practice.
